# Live Imaging of Immune Responses in Experimental Models of Multiple Sclerosis

**DOI:** 10.3389/fimmu.2016.00506

**Published:** 2016-11-21

**Authors:** Barbara Rossi, Gabriela Constantin

**Affiliations:** ^1^Section of General Pathology, Department of Medicine, University of Verona, Verona, Italy

**Keywords:** experimental autoimmune encephalomyelitis, T cell activation, regulatory T cells, two-photon microscopy

## Abstract

Experimental autoimmune encephalomyelitis (EAE) is the most common animal model of multiple sclerosis (MS), a chronic inflammatory autoimmune disease of the central nervous system (CNS) characterized by multifocal perivascular infiltrates that predominantly comprise lymphocytes and macrophages. During EAE, autoreactive T cells first become active in the secondary lymphoid organs upon contact with antigen-presenting cells (APCs), and then gain access to CNS parenchyma, through a compromised blood–brain barrier, subsequently inducing inflammation and demyelination. Two-photon laser scanning microscopy (TPLSM) is an ideal tool for intravital imaging because of its low phototoxicity, deep tissue penetration, and high resolution. In the last decade, TPLSM has been used to visualize the behavior of T cells and their contact with APCs in the lymph nodes (LNs) and target tissues in several models of autoimmune diseases. The leptomeninges and cerebrospinal fluid represent particularly important points for T cell entry into the CNS and reactivation following contact with local APCs during the preclinical phase of EAE. In this review, we highlight recent findings concerning the pathogenesis of EAE and MS, emphasizing the use of TPLSM to characterize T cell activation in the LNs and CNS, as well as the mechanisms of tolerance induction. Furthermore, we discuss how advanced imaging unveils disease mechanisms and helps to identify novel therapeutic strategies to treat CNS autoimmunity and inflammation.

## Introduction

Immune responses directed against self-antigens of the central nervous system (CNS) underlie several diseases, including multiple sclerosis (MS), neuromyelitis optica, and acute disseminated encephalomyelitis. MS is a chronic inflammatory demyelinating disease of the CNS affecting approximately 2–2.5 million people worldwide and leading to chronic progressive disability in the majority of cases (1). MS is heterogeneous both clinically and histopathologically, suggesting that different effector cells and molecular mechanisms are involved in the induction of tissue destruction (2). The most common form of MS, known as relapsing–remitting MS (RRMS), is associated with acute inflammatory episodes that reduce neurological function. RRMS patients may experience some recovery between relapses, but in 80% of cases, the disease evolves to a more progressive form, termed secondary progressive MS (SPMS). The latter is associated with a gradual loss of neurological function and ascending paralysis, both of which are believed to be less dependent on inflammation (3). Epidemiological and identical twin studies suggest that both genetics and environmental factors may play a role in disease pathogenesis (4, 5). Although the cause of MS is unknown, the presence of perivascular mononuclear cell infiltrates and demyelination suggest the disease is induced by an autoimmune response (4, 6, 7). Autoreactive T cell activation in MS is mainly associated with infection and is probably mediated by molecular mimicry, novel antigen presentation, bystander activation, or the coexpression of T cell receptors (TCRs) with different specificities (8–12). Antigen-presenting cells (APCs) containing myelin antigens were identified in the cervical lymph nodes (LNs) from MS patients, suggesting that the induction and propagation of autoimmune responses in MS starts at the periphery, within CNS draining LNs (13–15).

Several animal models have been developed to simulate clinical and histopathological patterns of demyelinating CNS inflammation, and these have been studied to determine the key steps in MS pathogenesis. The preferred animal model of MS is experimental autoimmune encephalomyelitis (EAE), which was introduced in 1925 by Koritschoner and Schweinburg, who induced spinal cord (SC) inflammation in rabbits by inoculation with human SC material (16). Since then, EAE has been induced by the immunization of susceptible animal strains, including primates and rodents, and has been shown to mimic several aspects of human MS (17). EAE can also be induced by the adoptive transfer of myelin-specific CD4^+^ T cell lines produced *in vitro*, proving that EAE is induced by an autoimmune response to myelin antigens (18). The shared clinical and histopathological features of EAE and MS suggest that both are autoimmune diseases induced by CNS-specific CD4^+^ autoreactive major histocompatibility complex class II (MHC-II) restricted T cells, which trigger a cascade of pathogenic events resulting in inflammation, demyelination, and neurodegeneration (19). Indeed, DR15 and DQw6 are the most important genetic susceptibility factors for MS, and myelin antigen-specific CD4^+^ MHC-II restricted T cells are more abundant in the blood and cerebrospinal fluid (CSF) of MS patients (20–23). During EAE, CNS-specific T cells are activated in the peripheral lymphoid organs and then migrate into the CNS (24). Inside the CNS, the T cells are reactivated by resident or infiltrating activated APCs, which present MHC-II associated peptides, resulting in inflammation, demyelination, and axonal damage.

Experimental autoimmune encephalomyelitis is considered an invaluable tool to study the activation of autoreactive T cells in the peripheral immune compartment, their migration into and reactivation within the CNS, and the subsequent induction of CNS inflammation. The activation and trafficking of immune system cells in EAE/MS is not fully understood. Recently, *in vivo* imaging techniques, such as two-photon laser scanning microscopy (TPLSM), have provided insights into the underlying disease mechanisms, leading to the development of novel therapeutic strategies to delay the progression of the disease. In this review, we discuss recent work on immune responses during EAE, highlighting the use of *in vivo* imaging to investigate T cell activation in lymphoid organs and the CNS and to study the basis of novel disease mechanisms.

## Immune Responses and Their Regulation During EAE

The most widely used protocol for EAE induction is currently based on the subcutaneous (sc) injection of an encephalitogenic peptide, which is emulsified in complete Freund’s adjuvant (CFA) containing mineral oil and *Mycobacterium tubercolosis* strain H37Ra, followed by intravenous (iv) administration of pertussis toxin as adjuvant. In the Swiss Jim Lambert (SJL) mouse (H-2s), EAE can be actively induced by immunization with CNS homogenate, proteolipid protein (PLP), myelin basic protein (MBP), or encephalitogenic epitopes of PLP (PLP_139–151_, PLP_178–191_), myelin oligodendrocyte protein (MOG_92–106_), or MBP (MBP_84–104_) in an emulsion with CFA (25). The disease follows a predictable clinical course, characterized by a prodromal period of 10–15 days followed by ascending paralysis beginning in the tail and hind limbs and progressing to the forelimbs concurrent with weight loss. In SJL mice, the disease involves a relapsing–remitting course of paralysis, allowing for mechanistic studies or immunomodulatory strategies in a relapsing autoimmune disease setting. MOG_35–55_ is a potent encephalitogenic peptide in C57BL/6 (H-2b) mice, and immunization with this peptide leads to chronic progressive disease. Generally, the resulting clinical EAE phenotype depends mainly on the antigen source and the genetic background of the animal species and strain.

Experimental autoimmune encephalomyelitis is a useful model for the investigation of immunological mechanisms responsible for the inflammatory autoimmune process in MS. During EAE, naïve autoreactive CD4^+^ T cells are activated in the secondary lymphoid organs and reach the CNS through the blood by extravasation across the blood–brain barrier (BBB) (26). Inside the CNS, the autoreactive CD4^+^ T cells are reactivated by resident or migrating APCs displaying CNS self-antigens, which are necessary for T-cell reactivation. This process is required for the pathogenesis of MS and EAE because it induces the production of soluble pro-inflammatory mediators (26). These molecules may trigger the recruitment of other inflammatory cells, including innate immune system cells, which are key contributors to demyelination and axonal damage (26).

Autoimmune diseases also reflect a failure to sustain immune tolerance to self and/or cross-reactive molecules. EAE models have contributed to the understanding of immunoregulatory processes during the pathogenesis of MS, and CD4^+^CD25^+^FoxP3^+^ regulatory T (T_reg_) cells represent the most efficient immunoregulatory cellular mechanism (27–30). Abnormalities in T_reg_ generation and function are considered a primary cause of autoimmune disease and other immunological disorders (31). These cells represent 5–10% of the CD4^+^ T lymphocytes in healthy adult mice and humans, and they have a specialized role in controlling both the innate and adaptive immune systems (32, 33). T_reg_ cells have been shown to modulate neuroinflammatory processes in several EAE studies. For example, Rag^−/−^ MBP-TCR transgenic mice develop spontaneous EAE and the depletion or inactivation of T_reg_ cells by the injection of an anti-CD25 monoclonal antibody results in a massive activation of autoreactive T cells, leading to more severe EAE and a delayed or abrogated recovery phase (34–36). In EAE induced by MOG_35–55_, both antigen-specific T-effector and T_reg_ cells differentiate and proliferate in the periphery before migrating to the CNS, with T_reg_ cells necessary for natural recovery after the disease peak observed in immunized EAE mice (36, 37). In both actively induced and passively induced EAE models, the accumulation and expansion of T_reg_ cells in the CNS correlates with recovery (36, 38). Dendritic cells (DCs) may be a major target of T_reg_-dependent immunoregulation in lymphoid organs during EAE and other animal models of autoimmunity (39–41).

## Visualization of T Cell Dynamics *In Vivo* by TPLSM – An Introduction

Two-photon laser scanning microscopy is advantageous because it achieves deep tissue penetration and high resolution with low phototoxicity, making it ideal to visualize immune system cells in living animals and to provide dynamic views of leukocytes (42, 43). TPLSM movies are acquired by rendering a raw time series of three-dimensional images with one or more fluorescent channels. Cell tracking requires the location of each cell within three-dimensional space at successive time points, and repeated imaging at defined time intervals can be used to determine a cell’s migratory path, velocity, motility, chemotactic index, and physical interactions with tissue components (43). The behavior of different cell types and the molecular mechanisms controlling their migration can be studied by using TPLSM to compare two or more experimental conditions (e.g., wild-type versus knockout animals, or animals with and without defined cell populations).

T cells are constantly in motion under physiological and pathological conditions, traveling over long distances in the blood, migrating into lymphoid or non-lymphoid organs through the endothelial wall, and then actively moving inside the tissues. TPLSM has been used extensively to study the motility behavior of these cells inside lymphoid and non-lymphoid tissues during autoimmune responses. For example, TPLSM has shown that following extravasation in the LNs, T cells initially move at high velocity, displaying a random walk or directed migration pattern to facilitate antigen surveillance (44–46). If migrating T cells come into direct contact with APCs in the vicinity of high endothelial venules (HEVs), their dynamic behavior undergoes changes, such as reduced motility or organization into clusters, leading to cell activation and proliferation (47, 48). T cell crawling on DCs has also been visualized in the LNs in the T cell area (49, 50). During viral infections, central memory CD8^+^ T cells may encounter antigen also in the peripheral areas in LNs presented by subcapsular sinus macrophages (51). Tolerance induction is accompanied by less stable DC/T cell interactions with shorter T cell/APC contact times, smaller DC/T cell clusters, and a rapid restoration of effector T cell motility (39–41, 52–54). Therefore, the detection of antigens and subsequent immune responses requires the long-range migration of cells, short-range communication, and direct cell–cell contact with APCs.

The reactivation of primed T cells in the CNS is necessary for the initiation of inflammatory responses and requires physical contact between T cells and local APCs. In the last decade, TPLSM has revealed the motility behavior of migrating T cells in the CNS and their reactivation inside the SC parenchyma, which is the main site of CNS inflammation in most EAE models (55–58). Importantly, T cells interact with APCs in the subarachnoid space during the early stages of EAE, and these contacts seem critical for T cell reactivation and expansion in the CNS (57, 59). TPLSM studies have been pivotal to the investigation of immune system cell behavior in the lymphoid tissue and CNS during autoimmune diseases, and the dynamic behavior of effector T cells and T_reg_ cells during the course of EAE is discussed in detail below.

## T Cell Dynamics in the LNs During EAE

In the absence of immunization, naïve T cells continuously migrate and scan antigen-presenting DC populations within the T cell zones of LNs (46, 49). Their fast and stochastic motility behavior facilitates a series of random encounters between antigen-specific T cells and DCs, thus favoring the initiation of potential adaptive immune responses (48, 60, 61). However, this apparently non-directed motility in the LNs is guided on rails, namely the fibroblastic reticular cells (FRCs), which are covered in chemokines and form tight networks in secondary lymphoid organs (62).

Dendritic cells in LNs upregulate their surface-processed antigen about 3 h after immunization, whereas migratory DCs appear after ~24 h (63, 64). Following immunization, interactions between antigen-specific T cells and antigen-loaded DCs in the LNs are prolonged compared to the steady state (65). They also undergo changes in motility involving initial transient serial encounters, followed by a phase of slowing and stable contacts, and then by a return to high motility (38, 48, 49, 66, 67). During antigen recognition, TCR ligation reduces or arrests T cell motility by sending stop signals through LFA-1 integrin, allowing the formation of stable T cell/APC conjugates and efficient activation (67–69). In agreement with TPLSM studies in other experimental models of autoimmunity, the induction of active EAE is followed within 20–24 h by a substantial reduction in the speed and motility coefficients of antigen-specific naïve T cells in response to antigen challenge in the draining LNs. Simultaneously, the arrest coefficient of these cells increases significantly, suggesting there is direct physical contact with DCs as confirmed by measuring individual T cell/DC interactions frame by frame (40, 41).

Dendritic cells are essential not only for the induction of antigen-specific immune responses but also for the maintenance of peripheral tolerance (70). *In vivo* studies have shown that immunity and tolerance both require the activation and clonal expansion of T cells following antigen-specific interactions between naïve T cells and APCs (71, 72). For example, an elegant TPLSM study using a tolerogenic immunoglobulin carrying the MOG_35–55_ peptide (Ig-MOG) has revealed T cell motility and T cell/DC interactions leading to T cell tolerance during EAE induced in 2D2 TCR transgenic mice (73, 74). This study showed that tolerized T cells had an activated T-helper (Th)2 phenotype resulting in the secretion of IL-4 and IL-5 cytokines, and their motility was reduced following tolerogen exposure similar to Th1 cells after immunization (74). However, the Th2 cells had higher migration velocities and took longer to exhibit changes in motility than Th1 cells, suggesting that both Th1 immunity and Th2 tolerance alter T cell migration following antigen recognition (74).

Although T cell activation during the initial phase of the immune response in the LNs has been studied in detail, less is known about the later stages of the disease. We used TPLSM to show that efficient antigen-dependent priming is also maintained during the later stages of active EAE induced by MOG_35–55_ (41). Our results showed that 7 days after immunization, during the preclinical phase of the disease, ovalbumin (OVA)-specific T cells moved faster, had a higher motility coefficient and a lower arrest coefficient than MOG_35–55_-specific T cells. MOG-specific effector T cells were more motile, had a lower arrest coefficient and a shorter contact time with DCs 7 days after immunization compared to MOG-specific T cells after 1 day, suggesting antigen-dependent activation is less effective. However, the number of stable contacts between T cells and DCs was higher after 7 days, suggesting that efficient antigen-dependent priming is also maintained during later phases of the immune response. Interestingly, the percentage of intermediate contacts was strongly reduced after 7 days, and the immune response was more polarized, with most T cells establishing transient or prolonged contacts with DCs. Adoptively transferred T cells appeared to lack contact with B cells and macrophages, suggesting that DCs provide the main mechanism for ongoing T cell activation during the late phase of the immune response during EAE. Activated MOG_35–55_-specific (but not OVA-specific) T cells tended to cluster, keeping close to each other without physical contact and swarming into organized spatial patterns, suggesting that proliferation and therefore clustering are necessary for the clonal expansion and maintenance of an immune response in the LNs. Sustained T cell activation and proliferation requires persistent antigen availability and the presentation of antigen by migratory DCs (75, 76). Subcutaneous immunization with an emulsion containing CFA in the EAE model allows the slow release and persistent presentation of antigen, favoring prolonged antigen-dependent T cell activation (77). In addition, some T cells may be primed in the LNs and may therefore be activated more efficiently due to the presence of chemokines and cytokines released during the 7 days of continuous antigen recognition. The presence of cytokines and chemokines is supported by the clustering of T cells, which suggests potential clonal expansion. Taken together, these observations suggest that antigen-dependent T cell activation is maintained during the late phase of the immune response, highlighting the importance of prolonged and sustained immune responses for the successful induction of autoimmunity.

## T_reg_ Cell-Dependent Dynamics in the LNs During EAE

Regulatory T cells are a subset of suppressor T cells that contribute to the maintenance of immunological homeostasis and self-tolerance (78). The mechanisms by which T_reg_ cells regulate immune responses are complex and incompletely understood, but there is a consensus that DCs are the major target of T_reg_ cells in lymphoid organs (39, 40, 79, 80). The contacts between T cells and DCs are highly regulated events influenced by the timing of activation, the signal strength and the inflammatory environment, so the regulation of these contact dynamics by T_reg_ cells is an essential component of the T cell activation process (60, 81, 82).

**Figure 1:**
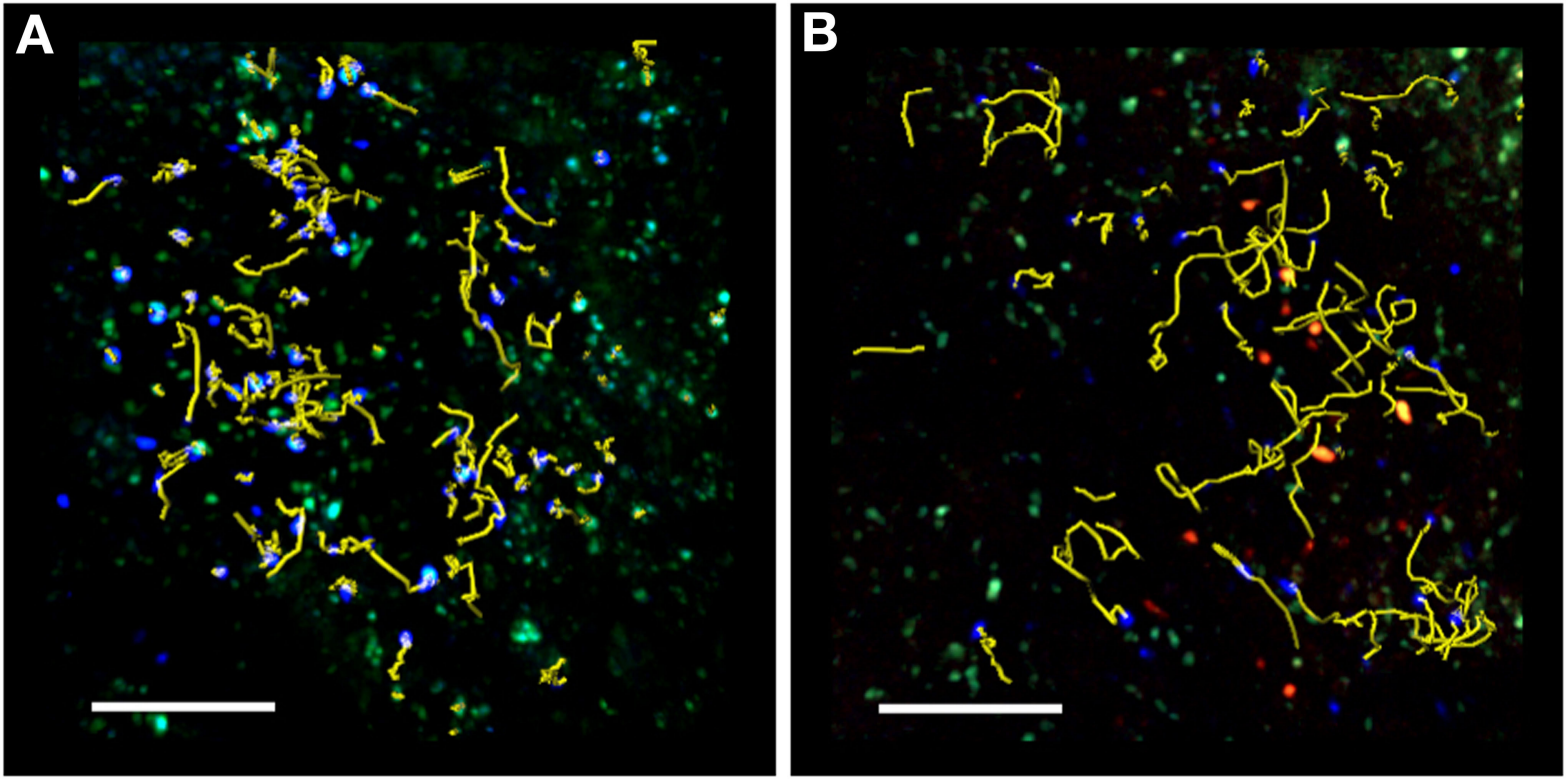
**TPLSM imaging of an exposed lymph node after MOG_35–55_ immunization**. Representative image of autoreactive T cells (blue) showing motility in the absence **(A)** or presence **(B)** of exogenous transplanted T_reg_ cells (red). TPLSM was performed using MHC-II-GFP transgenic mice to visualize APCs (green). For an intuitive assessment of cell motility, yellow cell tracks are displayed graphically to indicate the progression of naïve T cell movement during the imaging period. The resulting positional information in four dimensions (*xyz* coordinates and time) is the basis for all subsequent computational analysis. Two-photon multidimensional data analysis parameters revealed that during MOG_35–55_ immunization, T_reg_ cells modify the behavior of autoreactive T cells, increasing their motility and reducing their contact time with APCs in the peripheral LNs. Both figures are original. Scale bar = 100 micron.

Two-photon laser scanning microscopy studies during preclinical EAE have shown that the presence of T_reg_ cells increases the velocity and motility of T cells in draining LNs (as shown in Figure 1) (40). In addition, T_reg_ cells reduce the arrest coefficient of T cells in draining LNs by limiting the contact time between T cells and antigen-loaded DCs (40, 41). It is unclear how T_reg_ cells regulate T cell/DC interactions during EAE, but previous studies suggest that CTLA-4 may be necessary for the suppressive function of T_reg_ cells in the draining LNs after immunization (83). Supporting this hypothesis, previous TPLSM studies have shown that the presence of CTLA-4 on T_reg_ cells increases OVA-specific T cell motility and modulates the threshold for T cell activation (83). These studies of T cells specific for non-myelin antigens agree with TPLSM studies in EAE showing that T_reg_ cells control the priming of autoreactive T cells at very early stages of CNS disease by preventing persistent T cell interactions with DCs in the LNs (40, 41). Further support comes from previous studies showing that the presence of CTLA-4 on T_reg_ cells modulates CD80 and CD86 expression on DCs following antigen stimulation and suppresses the production of IL-6 and TNF-α by DCs (84–86). Finally, the analysis of explanted pancreatic LNs from non-obese diabetic mice has shown that persistent T_reg_/DC contacts prevent autoreactive T cell activation by DCs (39).

We recently used TPLSM imaging to investigate the negative modulation exerted by T_reg_ cells during the late phase of the immune response in the draining LNs in the EAE mouse. Imaging was carried out 7 days after immunization, when efficient antigen-dependent priming is maintained and the antigen-dependent clustering of effector T cells is associated with their high capacity for proliferation. We found that the presence of exogenous adoptively transferred T_reg_ cells increased the velocity and motility of antigen-specific T cells and reduced the contact time between T cells and DCs (41). Furthermore, T_reg_ cells inhibited the clustering of T cells and suppressed T cell proliferation *in vivo*, preferentially affecting Th1 rather than Th17 cell expansion during the late phase of the immune response (41). Previous studies have shown that the modulation of T cell proliferation by T_reg_ cells depends on the strength of the antigenic stimulus, whereas the modulation of chemokine production in DCs by T_reg_ cells does not (87). In this scenario, our data suggest that T_reg_ cells may inhibit pro-inflammatory cytokine/chemokine production and secretion by DCs, which is responsible for T cell clustering and may efficiently inhibit weaker antigen-specific T cell activation 7 days after immunization during the late phase of the immune response in EAE (41).

The role of P-selectin glycoprotein ligand 1 (PSGL-1), a trafficking receptor for leukocytes during inflammation, is not restricted to its function as an adhesion molecule. Indeed, its role extends to the regulation of immune responses (41, 88). PSGL-1 is associated with a DC tolerogenic phenotype in mice, and PSGL-1 deficiency has been correlated with increased T-cell proliferation and autoimmunity in several disease models including EAE, suggesting a role for PSGL-1 in tolerogenic mechanisms (41, 89–92). To investigate the function of PSGL-1 in the amelioration of EAE by T_reg_ cells, we recently used TPLSM to compare T cell dynamics in the presence of wild-type and PSGL-1 deficient T_reg_ cells in the LNs of CD11c-YFP-immunized mice. We found that the transfer of exogenous wild-type T_reg_ cells restored the motility of effector T cells by reducing the cell-cell contact time between effector T cells and DCs in immunological synapses during early-phase EAE (1 day after immunization) induced by MOG_35–55_. In this context, the presence of T_reg_ cells lacking PSGL-1 increased the velocity of T cells to the same extent as wild-type T_reg_ cells, confirming that PSGL-1 does not play any role during strong antigenic stimulation. At the later, preclinical phase of the disease (7 days after immunization) when the antigen-dependent activation of T cells declines but is still maintained in the LNs, T_reg_ cells reduced the duration of contact between MOG_35–55_ T cells and DCs, as well as their spatial clustering. However, the presence of exogenous transplanted PSGL-1 deficient T_reg_ cells was unable to inhibit the characteristic swarming into organized clusters and the resulting proliferation of MOG_35–55_ T effector cells (41). These data suggest that PSGL-1 is a key mediator of T_reg_-dependent suppression during the later stages of preclinical EAE, when antigenic stimulation in the LNs appears to be less efficient (41). The molecular mechanisms used by T_reg_ cells to modulate later-phase immune responses are still unknown, but we propose that PSGL-1 expression on the surface of T_reg_ cells could regulate DC activity by controlling contacts between the two cell types, as previously shown for LFA-1 (85). The molecular basis of such interactions may involve PSGL-1 on the surface of T_reg_ cells binding to L-selectin on the surface of DCs, although mature DCs express such low levels of this molecule that alternative ligands are more likely (93). PSGL-1 may also inhibit antigen-dependent chemokine secretion by DCs (80, 94). Therefore, the recruitment of inflammatory cells by LNs and the co-localization of antigen-bearing DCs with antigen-specific T cells suggests that the regulation of interactions between T_reg_ cells and DCs by PSGL-1 could modulate the local cytokine/chemokine profile in the LNs by reducing the clustering and activation of antigen-specific T cells (80, 94). In addition, PSGL-1 expressed on the surface of T_reg_ cells may sequester chemokines, such as CCL19/CCL21, and thus prevent interactions between newly arrived CCR7^+^ T cells and APCs during EAE (95, 96).

Another aspect of tolerance induction during EAE that remains unclear is the requirement for antigen-specific T_reg_ cells. The transfer of polyclonal T_reg_ cells ameliorated EAE in one study (97), but conflicting results were produced in another (98). However, two independent TPLSM studies have demonstrated that polyclonal naïve T_reg_ cells rapidly affect the motility of antigen-specific T cells, suggesting that antigenic specificity is not required by T_reg_ cells to modulate the development of EAE in the secondary lymphoid organs (40, 41). These results are supported by studies suggesting that T_reg_ cells may produce suppressive cytokines, thus overriding their antigen specificity (99, 100). Even so, studies in other experimental models support the need for antigen specificity, so further investigation is required to clarify whether antigen-specific suppression by T_reg_ cells is required in the LNs during EAE (39, 101, 102).

## The Dynamics of T Cell Activation in the Spinal Cord During EAE

**Figure 2:**
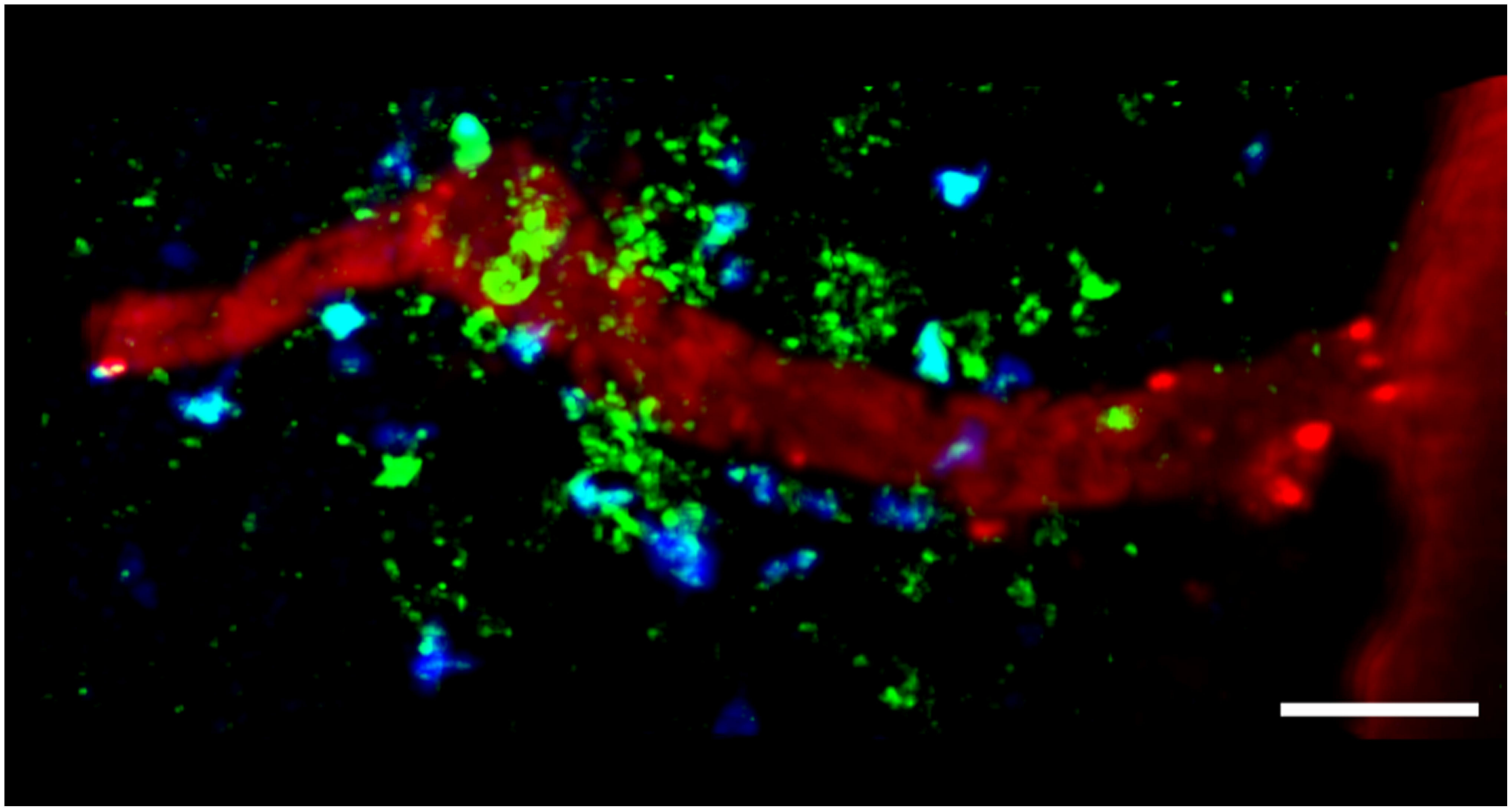
**TPLSM imaging of autoreactive T cells interacting with perivascular MHC-II^+^ APCs in the exposed spinal cord during EAE**. TPLSM was performed in MHC-II-GFP transgenic mice to visualize perivascular APCs (green) in contact with autoreactive T cells (blue). In order to investigate the types of APCs that can establish contacts with T cells, perivascular phagocytes were identified by intrathecally infused Texas Red-tagged dextran in MHC-II-GFP transgenic mice. These cells were located strategically around vessels, monitoring the environment with their cellular processes and sharing some morphological features with DCs, and others with macrophages. The merge of Texas Red and GFP positive cells provided evidence that perivascular phagocytes are functional APCs, expressing MHC-II determinants. The figure is original. Scale bar = 50 micron.

In organ-specific autoimmune disease models, pathogenic autoreactive T cells are activated in the periphery of the lymphoid organs before entering the target organ. For example, in an intravenous injection model of transfer EAE, encephalitogenic T cells activated *in vitro* migrate into lymphoid organs before they cross the BBB and enter the CNS (103). The reactivation of primed CD4^+^ T cells in the CNS requires interactions with local APCs during the initiation of an inflammatory response (Figure 2). The behavior of autoreactive T cells was investigated *in vitro* for the first time using a combination of TPLSM and fluorescence video microscopy to study SC slices obtained at the onset of EAE in a rat model (55). The results showed that MBP-specific effector T cells move through the CNS with two distinct migratory patterns: ~65% of the cells moved rapidly and randomly through the compact white and gray matter, whereas the remaining ~35% appeared tethered to a fixed point, suggesting the formation of immune synapses (55). Importantly, pretreatment of the SC tissue with neutralizing anti-MHC-II monoclonal antibodies significantly reduced the number of arrested autoreactive T cells (55). In contrast to the MBP-specific T cells, OVA-specific T cells did not form synapse-like contacts in the SC in this model but invaded the CNS and moved rapidly through the tissue (55). More recent *in vivo* TPLS studies based on laminectomy in the lumbar SC of a rat EAE model were used to visualize T cells within the meningeal areas and the adjacent white matter. These studies revealed that an intravenous infusion of soluble MBP during the acute EAE phase caused rapid antigen uptake by APCs in the CNS, resulting in the deceleration of MBP-specific T cells, a higher frequency of T cell immobilization, a stronger activating state, and the enhanced production of inflammatory cytokines (56). In this EAE model, BBB leakage allowed the uptake of circulating antigen that was quickly processed by CNS cells expressing MHC-II, which were preferentially located in meningeal areas and in the vicinity of vessels, i.e., the regions mainly infiltrated by T cells. However, effector and resting memory T cells specific for the non-CNS antigen OVA were also recruited to EAE lesions and moved there without contacting APCs. As shown for the MPB-specific T cells, the OVA-specific T cells were activated and arrested following the intravenous infusion of OVA, which increased the severity of disease symptoms. This suggested that effector T cells home toward acute EAE lesions and can be reactivated locally by exogenous antigen regardless of the specificity of the intruding T cell population (56). Thus, non-CNS antigens leaking into the chronically inflamed CNS through the bloodstream may trigger relapses in MS (56).

The MHC-II^+^ APCs located in the CNS during EAE mainly comprise perivascular macrophages and DCs, which induce the local reactivation of primed myelin-reactive T cells resulting in CNS inflammation and EAE progression (Figure 2) (55, 58). Flow cytometry showed that the most rapidly labeled phagocytes (30 min after the administration of intravenous antigen during the acute phase of EAE) share characteristics with macrophages, which express high levels of CD45, suggesting that these cells originate from the blood and are not resident cells (56). Later, the proteolytic digestion of the fluorescent antigen was observed in monocytes/macrophages, microglial cells, and B cells, suggesting these cells are involved in antigen presentation during EAE (56). The quantification of cell types in SC homogenates from mouse EAE models has confirmed the presence of CD11c^+^ DCs in the CNS, which are similar in phenotype and genotype to splenic DCs (104, 105). CNS-derived DCs can induce the proliferation of MOG-specific T cells, suggesting their importance in antigen presentation during EAE (105). Near-infrared imaging of DC transmigration has confirmed that these APCs can also migrate from the periphery into the CNS during EAE, and that their migration correlates with the severity of inflammation (106).

Recently, TPLSM imaging allowed the visualization of dynamic contacts between T cells and perivascular phagocytes (57, 107, 108). These results demonstrated that the leptomeninges and CSF are major routes for the migration of encephalitogenic T cells into the CNS during early phases of EAE in rats, and a site for T cell activation before the invasion of CNS parenchymal vessels (57, 107, 108). These studies showed that intravenously administrated MBP-specific T cells arrested on the leptomeningeal vessels and crawled intravascularly, preferentially against the blood flow (57). Following extravasation, T cells continue to scan the abluminal vascular surface and the underlying pial membrane, encountering phagocytes that effectively present antigens. These contacts induce the effector T cells to produce pro-inflammatory mediators, trigger tissue invasion and promote the formation of inflammatory infiltrates (57). Other reports confirm that T cells become activated in the perivascular space, but not within the vascular lumen, although previous *in vitro* studies have shown that endothelial cells can present antigen to adherent myelin-specific T cells (107, 109). In a MBP transfer EAE rat model, the comparison of highly immunogenic MBP-specific and modestly immunogenic MOG-specific T cells revealed that regardless of antigen specificity, the two cell types crawled with similar velocities in blood vessels (107). Once inside the CNS, the MBP-specific T cells moved within the leptomeningeal space with a lower velocity and mean square displacement than the MOG-specific T cells and had longer lasting contacts, whereas the MOG-specific T cells moved continuously in a straight line. However, activation signaling was not sufficient to completely arrest the highly immunogenic myelin-specific T cells in the leptomeningeal space, suggesting that contacts with APCs in the leptomeningeal area guide immigrant autoimmune T cells into the CNS parenchyma, rather than arresting them for extended periods of time (107). Before entering the CNS parenchyma, effector T cells traffic between the leptomeninges and CSF, with less adherent and activated T cells being flushed from the leptomeningeal network of collagen fibers by the CSF flow (108). TPLSM studies have shown that T cell adhesiveness to leptomeningeal structures is enforced by VLA-4 and LFA-1 integrins, CCR5/CXCR3-dependent signaling, and the antigenic stimulation of T cells in contact with leptomeningeal macrophages (108). However, T cells from the CSF fully retained their encephalitogenic potential and were able to reattach to the leptomeninges and invade the parenchyma (108). Together, these studies show that leptomeninges represent a key checkpoint for T-cell infiltration into the CNS during autoimmune inflammation or immune surveillance (57, 58, 108, 110).

It is unclear whether FRC structures like those in the LNs are present in the CNS parenchyma, but activated T cells can move in a similar manner in the CNS and LNs, suggesting analogous stromal structures are present in the CNS (111). Accordingly, TPLSM analysis in an experimental model of toxoplasmic encephalitis indicated the presence of an analogous reticular system associated with areas of parasite replication and local CNS inflammation, but not in normal brain tissues (111). CD4^+^ T-cell motility is actively promoted in the SC during EAE (112), and CD4^+^ T cells can gain access to CNS parenchyma and partially migrate along inflammation-induced extracellular matrix (ECM) structures, which are similar to those seen in LNs (112). These observations suggest that preexisting scaffolds guide lymphocyte migration in lymphoid tissues, but specialized structures are induced during CNS inflammation and guide T cell migration, potentially facilitating the screening of APCs and integrating relevant stimulatory, costimulatory, and regulatory signals (111–114).

The disease phase of effector T cell activation is crucial for the subsequent inflammatory process and the clinical disease (115). Using TPLSM, it was previously shown that the migration of myelin-specific T cells was mostly restricted to leptomeninges and perivascular areas during the preclinical phase and disease onset. However, during chronic phase of disease, T cells were spread throughout the white and gray matter (115). These studies have also shown that leptomeningeal phagocytes are responsible for the initial activation of encephalitogenic T cells in the CNS by mediating short cell–cell contacts with T cells (115). T cell activation process continues during disease progression and extends deeper in the CNS parenchyma through contacts with microglia and recruited phagocytes. However the initial activation during the preclinical phase of EAE seems to determine the clinical outcome of the autoimmune process (115). The visualization of effector T cells in their natural environment could also represent a useful tool to investigate the functional mechanisms behind therapeutic approaches during EAE. Using TPSLM, it was previously shown that administration of soluble myelin peptides or soluble intact MBP protein during the preclinical phase of diseases leads to a drastic reduction of antigen-specific T cell velocity in lymphoid peripheral organs (116). This efficiently prevented T cell migration into the CNS and abrogated CNS inflammation and disease development (116). In contrast, soluble antigen administration during established disease phase led to a strong aggravation of the clinical symptoms, increasing T cell activation and trafficking in the CNS (56).

## Tolerogenic Dynamics in the Spinal Cord During EAE

**Figure 3:**
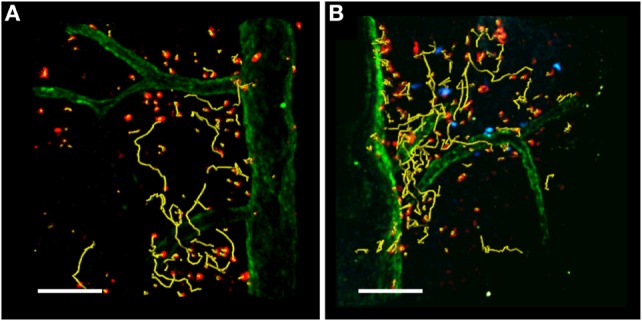
**TPLSM imaging of the exposed spinal cord after MOG_35–55_ immunization**. TPLSM representative dynamics (yellow tracks) of autoreactive Th1 cells (red) injected into C57Bl/6 EAE mice in the absence **(A)** or presence **(B)** of exogenous transplanted T_reg_ cells (blue). Spinal cord vessels (green) were labeled by the systemic injection of 525 nm non-targeted quantum dots. All figures are original. Scale bar = 100 micron.

The presence of T_reg_ cells during early-phase autoimmune diseases increases the motility of antigen-specific T cells in the LNs and reduces their contact time with DCs in lymphoid organs, contributing to the maintenance of self-tolerance (39–41). This view of preventive immune suppression in the LNs during EAE may be too simplistic because T_reg_ cells have also been detected in the inflamed CNS, where they can modify the motility behavior of antigen-specific T cells and their reactivation inside the CNS (Figure 3) (36, 37). The trafficking of T_reg_ cells to peripheral inflammation sites is necessary for their suppression of inflammation (117). In actively and passively induced EAE models, the accumulation of T_reg_ cells in the CNS correlates with restrained inflammation and recovery (36, 38). Also, T_reg_ cells are enriched in the CSF of relapsing–remitting MS patients (118) suggesting they migrate to the inflamed CNS and are potentially responsible for immunosuppression also during human disease.

Two-photon laser scanning microscopy was recently combined with the *in vivo* depletion of T_reg_ cells in a mouse EAE model to investigate the function of these cells in the CNS during EAE (119). Effector T cells were shown to move in confined trajectories in the meningeal SC of T_reg_ cell-depleted mice, with a reduced mean velocity and linearity index compared to the control non-depleted animals. In parallel, the stationary phase of T effector cells was increased in the absence of T_reg_ cells, indicating enhanced interactions with potential APCs within the inflamed SC during EAE associated with the increased capacity of T effector cells to proliferate in in the CNS. The suppression of T cell proliferation in the CNS of control mice with the normal complement of T_reg_ cells was associated with lower levels of IFNγ but similar levels of IL-17 compared to T_reg_ cell-depleted mice (119). These studies agree with previous data showing that CNS-derived T_reg_ cells obtained during recovery suppress the *in vitro* proliferation of CNS-derived T effector cells in response to antigen by limiting the activation of cells producing IFNγ but not those producing IL-17 (38). Furthermore, CNS-derived T_reg_ cells fail to control pathogenic T cells at peak EAE due to the local presence of IL-6 and TNF-α, potentially secreted by Th17 cells (37). Finally, the rapid progression of CNS autoimmunity can be prevented by T_reg_ cells *via* their ability to suppress IFNγ-mediated immune responses (120). Collectively, these results suggest that T_reg_ cells have a selective role at the site of CNS inflammation, depending on the local inflammatory setting, and the composition of the effector T cell population.

## Conclusion

TPLSM allows the visualization of cell motility and interaction dynamics in the immune system of living animals, revealing the behavior of effector T-cells in LNs and peripheral tissue sites during inflammation and autoimmunity. Efficient T cell motility in the SC parenchyma is regulated by CNS resident cells and the ECM. Therefore, inhibiting the movement of effector T cells or their interactions with local APCs, or potentiating the activity of T_reg_ cells in the CNS, may affect the development and progression of EAE/MS. The molecular mechanisms controlling the intra-tissue motility of activated T cell subsets, such as Th17 and Th1 cells in the CNS, and their interactions with local APCs could help to identify new treatments, ultimately for intrathecal administration, to complement existing systemic therapies for neurodegenerative autoimmune diseases. TPLSM could also be used to determine the molecular mechanisms by which T_reg_ cells regulate the immune response during EAE/MS, which is necessary to fully understand their role. Advanced imaging techniques will also help in the future to provide insights into the mechanisms controlling T cell activation during different disease phases, such as the chronic phase, and may lead to the development of novel therapeutic strategies based on the inhibition of stage-specific mechanisms to delay the progression of the disease.

## Author Contributions

BR and GC equally contributed to this work.

## Conflict of Interest Statement

The authors declare that the research was conducted in the absence of any commercial or financial relationships that could be construed as a potential conflict of interest.
